# A systematic review of the relationship between emotional intelligence and diabetes management

**DOI:** 10.1192/j.eurpsy.2022.897

**Published:** 2022-09-01

**Authors:** A. Pérez Fernández, P. Fernández-Berrocal, M.J. Gutiérrez-Cobo

**Affiliations:** 1 Faculty of Psychology. University of Málaga, Department Of Basic Psychology, Málaga, Spain; 2 Faculty of Psychology. University of Málaga, Department Of Developmental And Educational Psychology, Málaga, Spain

**Keywords:** Type 2 diabetes, Emotional Intelligence, Type 1 diabetes, Diabetes Management

## Abstract

**Introduction:**

Diabetes has been associated to affective disorders and mental health problems which complicate the management of the disease. Emotional intelligence (EI), or the ability to perceive, facilitate, understand and regulate emotions has shown to be a protective factor of emotional disorders in general population.

**Objectives:**

To evaluate the role of EI and EI training in the biological and psychological variables related to people with Type 1 and 2 diabetes.

**Methods:**

A systematic review was conducted in PubMed and Scopus database without time limitations, for studies examining the link between diabetes and EI. A total of 11 eligible studies were selected according to the inclusion criteria.

**Results:**

We divided the results into four sections: 1) EI and HbA1c, 2) EI training effects, 3) Differences in EI between persons with diabetes and without diabetes, and 4) EI and psychological adjustment and well-being. The results showed negative correlations between EI and HbA1C, positive effects of EI training on quality of life, anxiety and glycaemic control, no differences in EI between people with diabetes and healthy individuals and, finally, negative correlations between EI and different psychological variables such as diabetes-related anxiety and distress, and positive correlations with quality of life, well-being and marital satisfaction.

**Conclusions:**

EI appear to be a promising protective factor for biological and psychological variables in individuals with diabetes. This systematic review offers a starting point for a theoretical and practical understanding of the role played by EI in the management of diabetes. Limitation and future lines of investigations will be discussed.

**Disclosure:**

No significant relationships.

